# Impact in production costs resulting from the adoption of interventions to reduce antimicrobials in pig production in Europe: findings from EU-project AVANT

**DOI:** 10.1093/jacamr/dlag131

**Published:** 2026-07-14

**Authors:** João S. Afonso, Chantal M Morel, Anouschka Middelkoop, Joas Priem, Francesc Molist, Mathieu Gloaguen, Arnaud Buchet, Gunner Sørensen, Sally V Hansen, Jens P Nielsen, Luca Guardabassi, Jonathan Rushton

**Affiliations:** Department of Livestock and One Health, Institute of Infection, Veterinary & Ecological Sciences, University of Liverpool, Liverpool, UK; Department of Livestock and One Health, Global Burden of Animal Diseases Programme, University of Liverpool, Liverpool, UK; KPM Center for Public Management, University of Bern, Schanzeneckstrasse 1, Bern 3012, Switzerland; University Hospital Bonn, Institute for Hygiene and Public Health, Venusberg-Campus 1, Building 63, Bonn 53127, Germany; Schothorst Feed Research B.V., NA 8218, Lelystad, The Netherlands; Schothorst Feed Research B.V., NA 8218, Lelystad, The Netherlands; Schothorst Feed Research B.V., NA 8218, Lelystad, The Netherlands; Cooperl Innovation SAS, 1 Rue de la Gare, Plestan 22640, France; Cooperl Innovation SAS, 1 Rue de la Gare, Plestan 22640, France; SEGES Innovation P/S, Copenhagen, Denmark; SEGES Innovation P/S, Copenhagen, Denmark; Department of Veterinary and Animal Sciences, University of Copenhagen, Frederiksberg C 1870, Denmark; Department of Veterinary and Animal Sciences, University of Copenhagen, Frederiksberg C 1870, Denmark; Department of Livestock and One Health, Institute of Infection, Veterinary & Ecological Sciences, University of Liverpool, Liverpool, UK; Department of Livestock and One Health, Global Burden of Animal Diseases Programme, University of Liverpool, Liverpool, UK

## Abstract

**Objectives:**

Antimicrobial resistance is a major global health threat, with antimicrobial use recognized as a key driver. The post-weaning period is one of the most antimicrobial-intensive phases in pig production. This study assessed the impact on production costs of feed-based and immunomodulatory interventions designed to reduce antimicrobial use in European pig production.

**Materials and methods:**

Within the EU-funded AVANT project, we evaluated feed-based strategies (alfalfa diets and high-fibre/low-protein ‘secure’ feed) and immunomodulatory interventions (faecal filtrate transplantation and vaccination against Shiga-producing *Escherichia coli*). Using data from field trials and observational studies conducted in Denmark, the Netherlands and France, we estimated the additional production costs per kilogram of pigmeat associated with each intervention. Cost and production parameters were derived from InterPIG and EU datasets (2019–2023) and adjusted under alternative mortality scenarios (6%–24%).

**Results:**

Feed-based interventions for piglets accounted for a small share of total feed costs and increased production costs by <0.2% at EU level. Vaccination against Shiga-producing *E. coli* increased production costs by <1%. By contrast, faecal filtrate transplantation resulted in substantially higher costs under trial conditions, increasing production costs by 9%–11% depending on mortality assumptions. None of the interventions negatively affected pig performance metrics in the trials.

**Conclusions:**

Feed-based strategies and vaccination represent accessible and affordable options to reduce antimicrobial use in pig production in Europe. More innovative approaches such as faecal filtrate transplantation remain costly under experimental conditions but may become economically viable through regulatory approval, market development and economies of scale.

## Introduction

The selection of antimicrobial resistance (AMR) in bacterial pathogens may jeopardize our ability to manage infectious diseases. This escalating threat undermines decades of progress in medicine, veterinary care and food production, with major implications for public health, animal welfare and global food security. Recent modelling studies have projected that, without concerted global action, by 2050 AMR would become a leading cause of death worldwide,^1^ and be responsible for a 3.4% reduction in the global gross domestic product.^2^

The selection of AMR is intimately linked to the extent and pattern of bacterial exposure to antimicrobials. The use of antimicrobials applies selective pressure that can accelerate the emergence and spread of resistant strains.^3^ As such, managing the overuse and misuse of antimicrobials across sectors is widely recognized as a critical strategy for mitigating AMR and preserving the efficacy of existing antimicrobials.^4,5^

In EU livestock systems, pig production figures prominently among sectors with high antimicrobial use (AMU).^6^ Within pig production, enteric disorders induced by pathogenic *Escherichia coli*, in particular post-weaning diarrhoea (PWD), are a major indication for antimicrobial treatment.^7,8^ The stress of weaning, gut immaturity and dietary transition contribute to susceptibility in piglets, making interventions for enteric health a suitable candidate for reducing AMU.^9,10^

The AVANT—Alternatives to Veterinary Antimicrobials—project was a Horizon-2020 funded initiative designed to develop and test alternatives to antimicrobials for managing PWD in pigs under EU conditions.^11^ While the primary focus was on PWD management, the project also evaluated other interventions associated with AMU reduction in pig production in the French longitudinal database. Following results from pre-clinical studies, field trials conducted in AVANT tested the impact of feeding strategies and faecal filtrate transplantation (FFT) on pig health and performance. These included (i) a high-fibre diet based on alfalfa that promotes gut health, supporting beneficial microbial fermentation and reducing the proliferation of ETEC after weaning, and (ii) an on-farm FFT protocol that was designed to accelerate the establishment of a stable and resilient gut microbiota in newly weaned piglets, thereby enhancing colonization resistance against enteric pathogens and reducing disease susceptibility during the critical post-weaning period. In addition to the field trials, an extensive longitudinal dataset on pig production from a French integrator (henceforth referred to as the French database), encompassing animal health and production data and farm management and practices, was analysed to identify factors that were associated with improved animal health for gastrointestinal disorders. A vaccine against Shiga-producing *E. coli* and a high-fibre feed (hereafter referred to as ‘secure’ feed) were found to be associated with reduced AMU for gastroenteric disorders.^12^

However, biological efficacy alone is often insufficient to guarantee the adoption of the interventions. For farmers, the costs of changing production practices and/or implementing new tools must be weighed against the benefits.^13^

Given that economic considerations are central to farmers’ decision making and to the feasibility of large-scale implementation, this study focuses on quantifying the effect in production costs associated with these interventions. By doing so, it aims to support farmers, advisers and policymakers in understanding the financial trade-offs related to transitioning towards more cautious AMU in pig production.

## Materials and methods

Economic evaluations of interventions were performed using data from field trials and a large longitudinal database from a French integrator, which is described in more detail elsewhere.^12^ Only interventions that led to a statistically significant (*P* value <0.05) reduction in AMU were considered for this assessment. For the pre-weaning interventions to reduce post-weaning diarrhoea and AMU, FFT (Dutch trial) was included. For the post-weaning interventions, alfalfa as tested in Dutch and Danish trials, and a ‘secure feed’ as described in the French database study, were included, as well as an oral vaccine against Shiga-producing *E. coli* (Ecoporc^®^ Shiga, Ceva Santé Animale, Libourne, France).^12^ Although this vaccine targets Shiga-toxin producing *E. coli* (STEC) rather than enterotoxigenic *E. coli* (ETEC) responsible for PWD, its inclusion reflects the broader AVANT objective of identifying interventions associated with reduced AMU for gastroenteric disorders. The alfalfa-based diet consists of adding 5% alfalfa to creep feed administered to piglets. In the Dutch trial the alfalfa-based feed intervention was carried for 14 days after weaning, whereas in the Danish trial alfalfa-based diet was given until 29 days post-weaning. The ’secure’ feed protocol in the French farm contributing to the French database instructed a period of 21 days post-weaning on high-fibre feed. The on-farm FFT protocol consists of screening the faeces of lactating sows for pathogens, of which the pathogen-free faeces were pooled, diluted, centrifuged and microfiltrated. The resulting faecal filtrate was administered to neonatal piglets by an oral drencher on a daily basis during the first 6 days of life.^14^

There was heterogeneity in the study design and findings regarding the effects of the interventions in production and health parameters of post-weaning piglets. The different interventions, however, seemed to have little to no impact on production parameters. Also, piglets were not followed all the way to slaughter age (except for the Dutch trials), meaning that the benefits in performance under commercial conditions (such as days to slaughter) could not be compared among the interventions. As such, and to inform producers on the cost-side, the assessment of the economic implications of adopting the interventions focused on effect in production costs, without considering the eventual savings from reduced AMU and improved productivity.

Data on the different production parameters and production costs for pig production in the EU and individual EU countries were retrieved from available literature.^15^ For production parameters 2021 data were used (Table 1). For production costs, 2019 and 2023 data were used for the EU and for the included EU countries, respectively (Table 2). Data on the costs of each intervention were retrieved from project partners. For the alfalfa-based feed interventions data on feed costs were collected. For the FFT, data on the production of the filtrate (e.g. sample collection, laboratory costs, labour costs) were calculated as described in the Supplementary Materials (Table S1 and cost estimation model available as Supplementary data at *JAC* Online). Last, data on the vaccine price were collected. Data management was conducted in Microsoft Excel and data analysis and visualization were carried out in the R statistical environment.^17,18^

**Table 1.** Average production parameters for the EU and EU countries in 2021^15^

Country	Average liveweight at slaughter (kg)	Finishing FCR	Average cold carcass weight (kg)
Austria	122	2.85	95.60
Belgium	119	2.69	96.00
Denmark	119	2.58	89.60
EU	125	2.79	96.17
Finland	120	2.72	89.70
France	121	2.72	92.70
Germany	126	2.80	97.20
Hungary	111	2.97	87.80
Ireland	118	2.63	90.70
Italy	171	3.77	137.40
Netherlands	125	2.56	97.70
Spain	117	2.43	88.30
Sweden	125	2.78	91.30

**Table 2.** Average production costs for pig production (€/kg pigmeat) in the EU and EU countries^15,16^

Country	Year	Depreciation and finance costs (€/kg)	Feed costs (€/kg)	Labour costs (€/kg)	Other variable costs (€/kg)	Total costs (€/kg)
Austria	2023	0.55	1.36	0.22	0.25	2.38
Belgium	2023	0.25	1.24	0.13	0.24	1.86
Denmark	2023	0.30	1.22	0.17	0.25	1.94
EU	2019	0.23	0.99	0.15	0.27	1.64
Finland	2023	0.34	1.19	0.20	0.45	2.18
France	2023	0.31	1.26	0.15	0.33	2.04
Germany	2023	0.35	1.28	0.16	0.33	2.12
Hungary	2023	0.63	1.28	0.15	0.30	2.37
Ireland	2023	0.31	1.44	0.17	0.34	2.26
Italy	2023	0.52	1.50	0.15	0.28	2.46
Netherlands	2023	0.27	1.09	0.14	0.43	1.93
Spain	2023	0.22	1.37	0.11	0.30	2.00
Sweden	2023	0.39	1.30	0.16	0.22	2.08

The effect in production costs resulting from the intervention are expressed in €/kg pigmeat. The costs of the interventions were adjusted for the post-weaning to finishing production stage mortality. Mortality was informed by an InterPIG report, and set at 6%.^15^ Recognizing that the mortality in pig production can be higher depending on the context, the adjustment in costs of intervention was also done by doubling the mortality from 6% to 12%, and from 12% to 24%, to account for variation.

### Feed-related interventions

For feed interventions, the cost difference per pig was calculated as the difference between the total cost of feed consumed by the intervention group and the control group during the intervention period, as per equation (1).


(1) $$\begin{array}{c} \Delta FC_{i}=(\left(ADFI_{i}\;\times t_{i}\;\times \;FC_{i})\;-\;(ADFI_{c}\;\times \;t_{c}\;\times \;FC_{c}\right)) \end{array}$$

where $\Delta FC_{i}$ =  difference in the feed cost of intervention *i* (€), $ADFI_{i}$ =  average daily feed intake in intervention *i* (kg/day), $t_{i}\;$ *= * number of days intervention *i* feed is given, $FC_{i}$ =  feed costs (€/kg) of feed intervention *i,*  $ADFI_{c}$* =  *average daily feed intake in control (kg/day), $t_{c}\;$ *= * number of days control feed is given and $FC_{c}$ =  feed costs (€/kg) of control feed.

To account for mortality between weaning and slaughter, the cost per pig surviving to slaughter was adjusted according to equation (2).


(2) $$\begin{array}{c} \Delta FC_{i^{a}}=\Delta FC_{i}÷(1-m) \end{array}$$

where $\Delta FC_{i^{a}}$ =  adjusted difference in the feed cost of intervention *i* (€) and *m *=  post-weaning to finishing mortality (%).

Last, the cost per kg of pigmeat was calculated by dividing the adjusted cost per pig by the average cold carcass weight as per equation (3).


(3) $$\begin{array}{c} FIC_{i}=\Delta FC_{i^{a}}\;÷\;w_{c} \end{array}$$

where $FIC_{i}=\;$ costs per kg of pigmeat for ‘intervention’ feed *i* (€/kg pigmeat) and $w_{c}$ =  cold carcass weight (kg).

To contextualize the feed intervention within the pig production cycle, total feed consumption per pig was estimated by multiplying liveweight at slaughter by the finishing feed conversion ratio (FCR), thereby providing a scale for feed interventions relative to lifetime feed intake.

### Vaccination and faecal filtrate transplantation

For vaccination and FFT (non-feed-related interventions), the costs of the interventions were expressed as euros per pig head and also adjusted for mortality as per equation (4).


(4) $$\begin{array}{c} CI_{i^{a}}=C_{i}\;\;÷\;(1-m) \end{array}$$

where $CI_{i^{a}}$ =  adjusted cost of non-feed-related intervention *i* (€), $C_{i}$ =  costs of intervention *i* (€) and *m *=  post-weaning to finishing mortality (%).

To understand the added production costs resulting from adopting the non-feed-related interventions, the costs of the interventions ($ACI_{i})$ were divided by the carcass weight $(w_{c}$) (kg), as in equation (5).


(5) $$\begin{array}{c} NFIC_{i}=CI_{i^{a}}\;\;÷\;w_{c} \end{array}$$

where $NFIC_{i}$ =  costs per kg of pigmeat for non-feed-related intervention *i* (€/kg pigmeat).

The calculations on the costs per kg pigmeat for the FFT can be found in the Supplementary Materials (Table S1 and cost calculator model).

## Results

### Feed-related interventions

The field trials tested feed-based interventions (such as alfalfa) during specific periods of pig production. In the Dutch trial, pigs received ∼4.5 kg of intervention feed, while in the Danish trial they received ∼12 kg. This difference reflects the different durations of the two trials (Table 3).

**Table 3.** Total feed during interventions and price of each feed used

Data source	Feed intervention		Total feed (kg) per piglet	Feed price (€/kg)
Dutch trial	OAT^a^ (14 days PW^b^)		4.6	0.391
	ALF^c^ (14 days PW)		4.5	0.402
Danish trial	ALF^d^	Phase I (0–15 days PW)	3.8	0.396
		Phase II (16–29 days PW)	8.4	0.324
	Commercial	Phase I (0–15 days PW)	3.7	0.392
		Phase II (16–29 days PW)	8.3	0.316
French dataset	‘Secure’^c^ feed (21 days PW)		5.0	0.727
	Commercial (21 days PW)		5.0	0.685

^a^Diet with 4% oats

^b^Post-weaning

^c^Diet with 5% alfalfa

^d^Diets with higher fibre/low protein content

Using 2021 data, the estimated feed needed for producing one pig from birth until slaughter ranged from 284.3 kg in Spain to 644.7 kg in Italy. When we calculated the intervention feed as a percentage of total feed, the proportions were relatively small. For example, the 12 kg of alfalfa from the Danish trial would represent 4.27% of total feed in Spain (where pigs consume less feed overall). By contrast, the 4.5 kg from the Dutch trial would represent only 0.70% of total feed in Italy (where pigs consume much more feed overall) (Table 4).

**Table 4.** Total feed to produce one pig from birth to slaughter and the share (%) of ‘intervention’ feed in the total feed

Country	Total feed (kg)/pig produced	Dutch trial	Danish trial		French dataset
		ALF^a^ (%)	ALF^b^ Phase I (%)	ALF Phase II (%)	‘Secure’^c^ feed (%)
Austria	347.7	1.30	1.08	2.42	1.44
Belgium	320.1	1.41	1.17	2.62	1.56
Denmark	307.0	1.47	1.22	2.74	1.63
EU	348.8	1.29	1.08	2.41	1.43
Finland	326.4	1.38	1.15	2.57	1.53
France	329.1	1.37	1.14	2.55	1.52
Germany	352.8	1.28	1.06	2.38	1.42
Hungary	329.7	1.37	1.14	2.55	1.52
Ireland	310.3	1.45	1.21	2.71	1.61
Italy	644.7	0.70	0.58	1.30	0.78
Netherlands	320.0	1.41	1.17	2.63	1.56
Spain	284.3	1.59	1.32	2.95	1.76
Sweden	347.5	1.30	1.08	2.42	1.44

^a^Alfalfa diet in the Dutch trial.

^b^Alfalfa diet in the Danish trial.

^c^French secure low-protein/high digestible diet.

The highest difference in feed price (€/kg) was noticed in the French dataset, where the secure feed cost 0.042 euros more per kg compared with the commercial diet. The lowest difference was observed in the Danish trial Phase I (0.004 €/kg). The Dutch trial showed an intermediate difference with the alfalfa diet costing 0.011 €/kg more compared with the diet containing oats (which served as the control). Adjusting for mortality increased the differences proportionally across all interventions (Table 5).

**Table 5.** Feed price difference across the ‘intervention’ feed trials, unadjusted and adjusted for mortality

Trial/Data source		Feed price difference (€/kg)^a^			
		Unadjusted	6%	12%	24%
Danish	Phase I	0.0041	0.0044	0.0047	0.0054
	Phase II	0.0080	0.0085	0.0091	0.0105
Dutch		0.0114	0.0121	0.0130	0.0150
French dataset analysis		0.0420	0.0447	0.0477	0.0553

^a^The prices have been estimated unadjusted for mortality and also considering a 6%, 12% and 24% mortality

**Figure 1.**
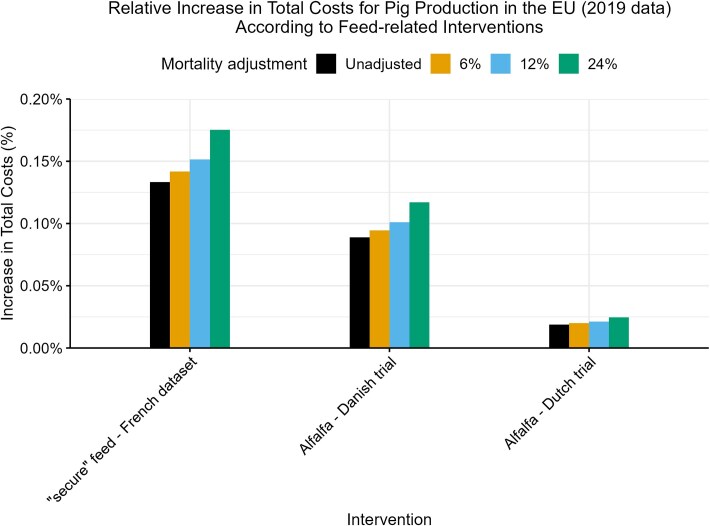
Relative increase in total production costs in pig production in the EU (2019 data) as a result of implementing the feed-related intervention.

Considering 2019 for the pig production costs in the EU, the relative increase in total costs resulting from the adoption of the feed-related interventions ranged from 0.019% for the Dutch trial using alfalfa (unadjusted) to 0.175% for the French dataset using secure feed when adjusted for 24% mortality. The Danish trial alfalfa intervention resulted in increases ranging from 0.089% (unadjusted) to 0.117% (adjusted for 24% mortality) (Figure 1 and Table S2 in the Supplementary Materials). Country-level results are presented in Table S2 in the Supplementary Materials.

### Vaccination and faecal filtrate transplantation

The FFT was estimated to cost 15.7 euros per piglet, unadjusted for mortality. The maximum price per piglet for FFT was 20.66 euros when adjusting for 24% mortality. Vaccinating each pig against *E. coli* cost 1.15 euros, unadjusted for mortality. When a 24% mortality rate was considered, vaccination costs per pig increased to 1.51 euros (Table 6).

**Table 6.** Price per piglet, unadjusted and adjusted for mortality, of the immunomodulatory interventions

Immunomodulatory intervention	Mortality adjustment (%)	Price (€) per piglet
FFT^a^	Unadjusted	15.70
	6	16.70
	12	17.84
	24	20.66
Vaccine (*E. coli*)	Unadjusted	1.15
	6	1.22
	12	1.31
	24	1.51

^a^Faecal filtrate transplantation

Considering the carcass weight (2021 data) the costs per kg of pigmeat per pig slaughtered, unadjusted for mortality, ranged from 0.179 euros in Hungary to 0.114 euros in Italy. The costs per kg of pigmeat per pig slaughtered for the *E. coli* vaccine were highest in Denmark, Finland, Hungary, Ireland, Spain and Sweden—0.013 euros—and lowest in Italy—0.008 euros (Table 7).

**Table 7.** Cost per kg of pigmeat per pig slaughtered of the immunomodulatory interventions across some EU countries and the EU (2021 data)

Country	Year	Average cold carcass weight (kg)	FFT^a^	*E. coli* vaccine
Austria	2021	95.60	€ 0.164	€ 0.012
Belgium	2021	96.00	€ 0.164	€ 0.012
Denmark	2021	89.60	€ 0.175	€ 0.013
EU	2021	96.17	€ 0.163	€ 0.012
Finland	2021	89.70	€ 0.175	€ 0.013
France	2021	92.70	€ 0.169	€ 0.012
Germany	2021	97.20	€ 0.162	€ 0.012
Hungary	2021	87.80	€ 0.179	€ 0.013
Ireland	2021	90.70	€ 0.173	€ 0.013
Italy	2021	137.40	€ 0.114	€ 0.008
Netherlands	2021	97.70	€ 0.161	€ 0.012
Spain	2021	88.30	€ 0.178	€ 0.013
Sweden	2021	91.30	€ 0.172	€ 0.013

^a^Faecal filtrate transplantation

**Figure 2.**
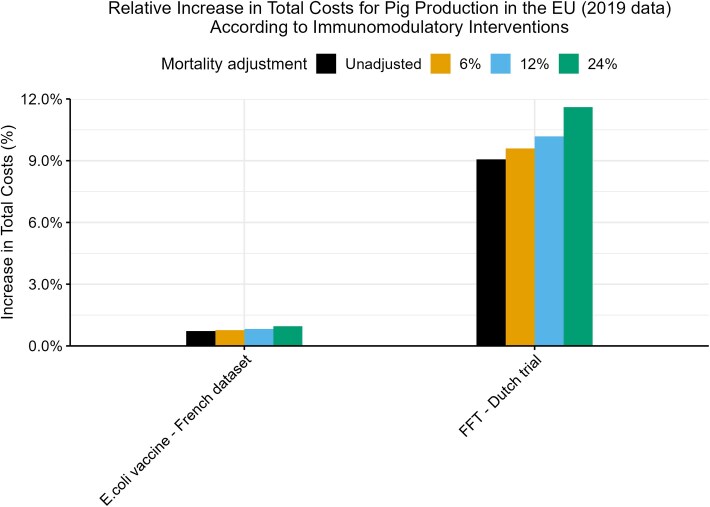
Relative increase in total production costs in pig production in the EU (2019 data) as a result of implementing the immunomodulatory interventions.

The adoption of the FFT adjusted for 24% mortality rate, had the highest relative increase in total production costs in the EU (2019 data), 11.6%. When unadjusted for mortality, it led to an increase of 9.1% in total production costs. When adopted, the vaccine against *E. coli* increased the total costs of pig production by <1% (from 0.73% when unadjusted to 0.95% when adjusted) (Figure 2 and Table S.3). Country-level results are presented in Table S3 in the Supplementary Materials.

## Discussion

Decision making in livestock farming is a complex process, driven by different factors and considering different data, with economics playing a central role.^19,20^ This study quantified the impact in production costs associated with adopting interventions designed to prevent PWD and reduce AMU in EU pig production. Whereas feed-based interventions and vaccination against Shiga-producing *Escherichia coli* led to only minor increases in production costs (<1%), implementation of FFT was associated with a much larger cost rise (11.6%). It should be noted that the high-cost rise regarding FFT reflect current trial-scale implementation and are therefore likely to overestimate the costs that could be achieved following regulatory approval, standardization of production processes, and the expansion to commercial-scale supply. Given its current state of technological development, the FFT must be prepared ‘in-house’, implying laboratory costs to analyse the safety of the faeces of the donors and preparation of large volumes of faecal filtrate. The handling of individual piglets for the oral administration of the filtrate is also burdensome in terms of labour costs. An additional hurdle to the uptake of such technology might be acceptance. In a survey conducted within AVANT with consumers, pig veterinarians and pig producers across five countries to understand the perception of different technologies aimed at reducing AMU in pig production, FFT was among the technologies with the lowest levels of acceptance.^21^ It should be noted that while FFT, alfalfa-based diets, and secure feed directly target PWD prevention, the STEC vaccine represents a complementary strategy addressing a different *E. coli* pathotype. Its inclusion reflects the pragmatic reality that comprehensive AMU reduction requires multiple interventions targeting different disease challenges within the same production system.

These findings highlight the accessibility and affordability of interventions that can reduce AMU with minimal impact on the cost structure. This point is further reinforced by recognizing that the additional costs have not been weighed against the avoided expenditures from fewer treatments and the potential broader benefits of improved animal health at early stages of development. Having available data to evaluate the cost–benefit of these interventions could help to strengthen the business-case for adopting these interventions, and examples exist in the literature in which changes in production practices have proved to provide return on investment to the farmers and additional benefits for other stakeholders across the value chain. Niemi *et al.*, using a value-chain approach, concluded that interventions to control production diseases in intensive pig farming can generate benefits for farmers through improved productivity and profitability while also delivering advantages to consumers through safer and more sustainable products.^21^ Postma *et al.* showed that enhanced biosecurity, vaccination and herd management measures substantially reduced AMU while maintaining or improving technical performance parameters, indicating that the economic impacts at farm level can be positive even when modest additional costs are incurred.^22,23^

It is important to note that this study focused on monetary aspects (costs), and that these alone are insufficient for understanding the adoption of antimicrobial reduction interventions, as other non-monetary factors, which were beyond the scope of this analysis, play an important role. Different interventions vary substantially in their efficacy at reducing AMU, their safety profiles, regulatory requirements and practical feasibility at farm level. For example, vaccination against Shiga-producing *E. coli* is already commercialized and widely available across the EU, which may be explained by its cost-efficacy and its straightforward integration into herd health programmes.^24^ By contrast, FFT has an experimental nature in livestock production and faces regulatory barriers that currently compromise its accessibility and any attempt at marketization. Farmer perceptions, ease of adoption, compatibility with existing production systems and consumer acceptance are equally important factors that influence the real-world uptake of these interventions. A comprehensive evaluation framework should therefore integrate economic, regulatory, safety and socio-behavioural dimensions to guide evidence-based decision making.^25^

Beyond farm-level considerations, the broader market and value-chain context also influences the feasibility of adopting AMR-mitigation interventions. When examining the economic viability of antimicrobial reduction strategies from a value-chain perspective, information on the added costs resulting from interventions might already set the tone for incentivizing changes in production practices. According to literature consumers are willing to pay more for pork produced with lower levels of antimicrobials.^26–28^ Such attitude opens an opportunity for the added costs resulting from the adoption of interventions to be shared across the whole value chain. In addition, and even though pig production sector is heterogenous across the EU, the high level of vertical integration in some of the main European pig producers enables such changes to be widely adopted from a stronger bargaining position.

While market mechanisms such as consumer willingness-to-pay and vertical integration may facilitate the adoption of interventions to mitigate antimicrobial resistance (AMR), a comprehensive economic evaluation must also account for the broader societal benefits that extend beyond direct market transactions. Antimicrobial resistance is a cross-species, multidimensional and intersectoral problem, meaning that mitigation actions aimed at reducing its likely occurrence generate positive externalities (an affect resulting from an economic activity effecting people who are not directly involved in it) beyond the population directly benefiting from such actions.^29^ Future work should integrate stochastic cost–benefit analyses of these interventions at farm level, and also integrate the externalities generated as a result of implementing these interventions. Working in silos with a One Health problem such as AMR might fail to provide the full picture in relation to effect of useful interventions. Roth *et al.* give a good example of such phenomena, highlighting that a livestock vaccination programme against brucellosis was not cost-beneficial when considering solely the animal population, but went on to provide significantly return on investment when considering also the human population due to reduced human cases of brucellosis and its associated costs.^30,31^

The pig sector is diverse in terms of infrastructure, performance, epidemiological landscape.^32^ Results from this study have been produced through challenging the costs of the interventions against the average production costs in EU pig production. As such, extrapolation of these findings to specific production contexts should be undertaken with caution, and context-specific investigations are warranted before inferring comparable outcomes.

### Conclusion

The study explored the cost of introducing accessible, affordable and existing interventions to reduce AMU for post-weaning diarrhoea in pig production, without considering the benefits (e.g. improved pig performance, reduced veterinary costs) and non-monetary factors (e.g. regulatory issues, stakeholder acceptance). Feed-based and vaccination measures can be implemented with only marginal increases in production costs (<1%), making them realistic tools for achieving AMU reductions without jeopardizing farm profitability. More innovative approaches, such as FFT, currently entail higher costs at trial scale (up to 11.6% increase in production costs) but could become affordable if regulatory barriers are removed and production reaches commercial scale.

## Supplementary Material

dlag131_Supplementary_Data

## Supplementary data

Tables S1–S3 are available as Supplementary data at *JAC* Online.

## References

[1] O’Neill J . Tackling Drug-Resistant Infections Globally: Final Report and Recommendations. Review on Antimicrobial Resistance, 2016.

[2] Jonas O, Irwin A, Berthe FCJ et al Drug-resistant Infections: A Threat to our Economic Future. World Bank Group, 2017.

[3] Salam MA, Al-Amin MY, Salam MT et al Antimicrobial resistance: a growing serious threat for global public health. Healthcare 2023; 11: 1946. 10.3390/healthcare11131946 37444780 PMC10340576

[4] OECD . Embracing a One Health framework to fight antimicrobial resistance. OECD Health Policy Studies, 2023.

[5] EMA Committee for Medicinal Products for Veterinary Use (CVMP) and EFSA Panel on Biological Hazards (BIOHAZ), Murphy D, Ricci A et al EMA and EFSA joint scientific opinion on measures to reduce the need to use antimicrobial agents in animal husbandry in the European Union, and the resulting impacts on food safety (RONAFA). EFSA J 2017; 15: e04666. 10.2903/j.efsa.2017.4666 32625259 PMC7010070

[6] O'Neill L, Rodrigues da Costa M, Leonard FC et al Quantification, description and international comparison of antimicrobial use on Irish pig farms. Porcine Health Manag 2020; 6: 30. 10.1186/s40813-020-00166-y 33062293 PMC7549222

[7] Dewulf J, Joosten P, Chantziaras I et al Antibiotic use in European pig production: less is more. Antibiotics 2022; 11: 1493. 10.3390/antibiotics11111493 36358148 PMC9686698

[8] Luppi A, D'Annunzio G, Torreggiani C et al Diagnostic approach to enteric disorders in pigs. Animals 2023; 13: 338. 10.3390/ani13030338 36766227 PMC9913336

[9] Han X, Hu X, Jin W et al Dietary nutrition, intestinal microbiota dysbiosis and post-weaning diarrhea in piglets. Animal Nutrition 2024; 17: 188–207. 10.1016/j.aninu.2023.12.010 38800735 PMC11126776

[10] Canibe N, Højberg O, Kongsted H et al Review on preventive measures to reduce post-weaning diarrhoea in piglets. Animals 2022; 12: 2585. 10.3390/ani12192585 36230326 PMC9558551

[11] AVANT Consortium . AVANT—Alternatives to Veterinary Antimicrobials. https://avant-project.eu/ (Accessed 15 January 2026).

[12] Buchet A, Samson V, Riera B et al Effet des facteurs d’élevage et des changements de pratiques sur l’utilisation des antibiotiques à visée digestive chez le porcelet. Journees de la Recherche Porcine 2025; 57: 279–84.

[13] Rushton J . The Economics of Animal Health and Production. Cabi, 2009.

[14] Middelkoop A, Priem J, Larsen C et al 10. Fecal filtrate transplantation and dietary fibre supplementation as alternatives to veterinary antimicrobials. Animal-Science Proceedings 2025; 16: 311–2. 10.1016/j.anscip.2025.07.011

[15] InterPIG . 2021 Pig Cost of Production in Selected Countries. Agriculture and Horticulture Development Board (AHDB), 2022.

[16] Microsoft Corporation . Microsoft Excel. 2016. https://office.microsoft.com/excel.

[17] R Core Team . R: A Language and Environment for Statistical Computing. 2020.

[18] Taramuel-Taramuel JP, Montoya-Restrepo IA, Barrios D. Drivers linking farmers’ decision-making with farm performance: a systematic review and future research agenda. Heliyon 2023; 9: e20820. 10.1016/j.heliyon.2023.e20820 37867840 PMC10585299

[19] Hayden MT, Mattimoe R, Jack L. Sensemaking and the influencing factors on farmer decision-making. J Rural Stud 2021; 84: 31–44. 10.1016/j.jrurstud.2021.03.007

[20] Filoklis P . Summary report on opinion surveys targeting end users. *Zenodo* 2022. 10.5281/zenodo.7273577.

[21] Niemi J, Bennett R, Clark B et al A value chain analysis of interventions to control production diseases in the intensive pig production sector. PLoS ONE 2020; 15: e0231338. 10.1371/journal.pone.0231338 32267875 PMC7141678

[22] Postma M, Vanderhaeghen W, Sarrazin S et al Reducing antimicrobial usage in pig production without jeopardizing production parameters. Zoonoses Public Health 2017; 64: 63–74. 10.1111/zph.12283 27362766

[23] EU PiG Innovation Group . Technical Report—Health Management 2018; https://projectblue.blob.core.windows.net/media/Default/EUPiG/Yr1_Health.pdf

[24] Baudoin F, Hogeveen H, Wauters E. Reducing antimicrobial use and dependence in livestock production systems: a social and economic sciences perspective on an interdisciplinary approach. Front Vet Sci 2021; 8: 584593. 10.3389/fvets.2021.584593 33816582 PMC8012488

[25] Paudel B, Kolady D, Grebitus C et al Consumers’ willingness to pay for pork produced with different levels of antibiotics. Q Open 2022; 2: qoac001. 10.1093/qopen/qoac001

[26] Bradford H, McKernan C, Elliott C et al Consumers’ perceptions and willingness to purchase pork labelled ‘raised without antibiotics’. Appetite 2022; 171: 105900. 10.1016/j.appet.2021.105900 34968563

[27] Kozera-Kowalska M, Uglis J. Consumer attitudes and purchase intentions for antibiotic-free pork in Poland: an empirical study. Eur Res Stud J 2024; 27: 295–311. 10.35808/ersj/3518

[28] Leal JR, Conly J, Henderson EA et al How externalities impact an evaluation of strategies to prevent antimicrobial resistance in health care organizations. Antimicrob Resist Infect Control 2017; 6: 53. 10.1186/s13756-017-0211-2 28588766 PMC5457558

[29] Babo Martins S, Sucena Afonso J, Fastl C et al The burden of antimicrobial resistance in livestock: a framework to estimate its impact within the global burden of animal diseases programme. One Health 2024; 19: 100917. 10.1016/j.onehlt.2024.100917 39497949 PMC11533088

[30] Roth F, Zinsstag J, Orkhon D et al Human health benefits from livestock vaccination for brucellosis: case study. Bull World Health Organ 2003; 81: 867–76. 14997239 PMC2572379

[31] Mateos G, Corrales N, Talegón G et al Pig meat production in the European Union—27: current status, challenges, and future trends. Anim Biosci 2024; 37: 755–74. 10.5713/ab.23.0496 38606453 PMC11016692

[32] InterPIG . InterPIG Dashboard. https://interpig.org/dashboard (Accessed Jan 2025).

