# Supplementary material for: Impact in production costs resulting from the adoption of interventions to reduce antimicrobials in pig production in Europe: findings from EU-project AVANT
Source: JAC Antimicrob Resist. 2026 Jul 14;8(4):dlag131. doi: 10.1093/jacamr/dlag131 (PMC13366534; doi:10.1093/jacamr/dlag131)
Supplement: dlag131_Supplementary_Data [file dlag131_supplementary_data.zip › Sup_mat_Eco_implications_AVANT_trials_final.docx]

Supplementary Material

# Faecal filtrate transplantation costs

Table S. 1. Different costs for producing faecal filtrate and price per kg pigmeat for faecal filtrate transplantation

| **Costs** | |
| --- | --- |
| **SM buffer** | |
| Ingredient cost (€/L) | 1.51 |
| Labor cost (€/L) | 1.73 |
| **Total costs (€/L)** | **3.24** |
|  |  |
| **Fecal collection labor** | |
| **Total costs (€)** | **34.32** |
| **Cost per liter fecal filtrate (€/L)** | **2.12** |
|  |  |
| **Fecal pathogen testing** | |
| **Total cost pathogen testing (€**) | **5405.40** |
| **Costs per liter fecal filtrate (€/L)** | **333.28** |
|  |  |
| **Filtration** | |
| Labor cost dilution and gross filtration | 20.80 |
| Labor cost centrifugation | 135.20 |
| Labor cost filtration | 168.11 |
| **Total labor costs (€)** | **324.11** |
| **Labor costs per liter filtrate (€/L)** | **19.98** |
| Depreciation filtration system per FFT batch (€) | 90.59 |
| Depreciation filtration system per liter fecal filtrate (€) | 5.59 |
| Depreciation membrane per FFT batch (€) | 222.46 |
| Depreciation membrane per liter fecal filtreate (€) | 13.72 |
| **Total depreciation per FFT batch (€)** | **313.05** |
| **Total depreciation per liter fecal filtrate (€)** | **19.30** |
|  |  |
| **Inoculation** | |
| Fecal filtrate cost per piglets (€/piglet) | 13.62 |
| **Labor cost per piglet (€/piglet)** | **2.08** |
| **Total costs per piglet (€/piglet)** | **15.70** |

# EU and Country-level results

Table S. 2. Relative increase in pig production costs in EU countries and in the EU as a result of adopting feed-related interventions

| Country | Intervention | Trial/source | Mortality adjustment | Relative increase in total costs |
| --- | --- | --- | --- | --- |
| Austria | Alfalfa | Dutch trial | unadjusted | 0.01% |
| Belgium | Alfalfa | Dutch trial | unadjusted | 0.02% |
| Denmark | Alfalfa | Dutch trial | unadjusted | 0.02% |
| EU | Alfalfa | Dutch trial | unadjusted | 0.02% |
| Finland | Alfalfa | Dutch trial | unadjusted | 0.02% |
| France | Alfalfa | Dutch trial | unadjusted | 0.02% |
| Germany | Alfalfa | Dutch trial | unadjusted | 0.01% |
| Hungary | Alfalfa | Dutch trial | unadjusted | 0.01% |
| Ireland | Alfalfa | Dutch trial | unadjusted | 0.01% |
| Italy | Alfalfa | Dutch trial | unadjusted | 0.01% |
| Netherlands | Alfalfa | Dutch trial | unadjusted | 0.02% |
| Spain | Alfalfa | Dutch trial | unadjusted | 0.02% |
| Sweden | Alfalfa | Dutch trial | unadjusted | 0.02% |
| Austria | Alfalfa | Dutch trial | 6% | 0.01% |
| Belgium | Alfalfa | Dutch trial | 6% | 0.02% |
| Denmark | Alfalfa | Dutch trial | 6% | 0.02% |
| EU | Alfalfa | Dutch trial | 6% | 0.02% |
| Finland | Alfalfa | Dutch trial | 6% | 0.02% |
| France | Alfalfa | Dutch trial | 6% | 0.02% |
| Germany | Alfalfa | Dutch trial | 6% | 0.02% |
| Hungary | Alfalfa | Dutch trial | 6% | 0.02% |
| Ireland | Alfalfa | Dutch trial | 6% | 0.02% |
| Italy | Alfalfa | Dutch trial | 6% | 0.01% |
| Netherlands | Alfalfa | Dutch trial | 6% | 0.02% |
| Spain | Alfalfa | Dutch trial | 6% | 0.02% |
| Sweden | Alfalfa | Dutch trial | 6% | 0.02% |
| Austria | Alfalfa | Dutch trial | 12% | 0.01% |
| Belgium | Alfalfa | Dutch trial | 12% | 0.02% |
| Denmark | Alfalfa | Dutch trial | 12% | 0.02% |
| EU | Alfalfa | Dutch trial | 12% | 0.02% |
| Finland | Alfalfa | Dutch trial | 12% | 0.02% |
| France | Alfalfa | Dutch trial | 12% | 0.02% |
| Germany | Alfalfa | Dutch trial | 12% | 0.02% |
| Hungary | Alfalfa | Dutch trial | 12% | 0.02% |
| Ireland | Alfalfa | Dutch trial | 12% | 0.02% |
| Italy | Alfalfa | Dutch trial | 12% | 0.01% |
| Netherlands | Alfalfa | Dutch trial | 12% | 0.02% |
| Spain | Alfalfa | Dutch trial | 12% | 0.02% |
| Sweden | Alfalfa | Dutch trial | 12% | 0.02% |
| Austria | Alfalfa | Dutch trial | 24% | 0.02% |
| Belgium | Alfalfa | Dutch trial | 24% | 0.02% |
| Denmark | Alfalfa | Dutch trial | 24% | 0.02% |
| EU | Alfalfa | Dutch trial | 24% | 0.02% |
| Finland | Alfalfa | Dutch trial | 24% | 0.02% |
| France | Alfalfa | Dutch trial | 24% | 0.02% |
| Germany | Alfalfa | Dutch trial | 24% | 0.02% |
| Hungary | Alfalfa | Dutch trial | 24% | 0.02% |
| Ireland | Alfalfa | Dutch trial | 24% | 0.02% |
| Italy | Alfalfa | Dutch trial | 24% | 0.01% |
| Netherlands | Alfalfa | Dutch trial | 24% | 0.02% |
| Spain | Alfalfa | Dutch trial | 24% | 0.02% |
| Sweden | Alfalfa | Dutch trial | 24% | 0.02% |
| Austria | Alfalfa | Danish trial | unadjusted | 0.06% |
| Belgium | Alfalfa | Danish trial | unadjusted | 0.08% |
| Denmark | Alfalfa | Danish trial | unadjusted | 0.08% |
| EU | Alfalfa | Danish trial | unadjusted | 0.09% |
| Finland | Alfalfa | Danish trial | unadjusted | 0.07% |
| France | Alfalfa | Danish trial | unadjusted | 0.07% |
| Germany | Alfalfa | Danish trial | unadjusted | 0.07% |
| Hungary | Alfalfa | Danish trial | unadjusted | 0.07% |
| Ireland | Alfalfa | Danish trial | unadjusted | 0.07% |
| Italy | Alfalfa | Danish trial | unadjusted | 0.04% |
| Netherlands | Alfalfa | Danish trial | unadjusted | 0.07% |
| Spain | Alfalfa | Danish trial | unadjusted | 0.08% |
| Sweden | Alfalfa | Danish trial | unadjusted | 0.07% |
| Austria | Alfalfa | Danish trial | 6% | 0.07% |
| Belgium | Alfalfa | Danish trial | 6% | 0.08% |
| Denmark | Alfalfa | Danish trial | 6% | 0.09% |
| EU | Alfalfa | Danish trial | 6% | 0.09% |
| Finland | Alfalfa | Danish trial | 6% | 0.08% |
| France | Alfalfa | Danish trial | 6% | 0.08% |
| Germany | Alfalfa | Danish trial | 6% | 0.07% |
| Hungary | Alfalfa | Danish trial | 6% | 0.07% |
| Ireland | Alfalfa | Danish trial | 6% | 0.07% |
| Italy | Alfalfa | Danish trial | 6% | 0.04% |
| Netherlands | Alfalfa | Danish trial | 6% | 0.08% |
| Spain | Alfalfa | Danish trial | 6% | 0.08% |
| Sweden | Alfalfa | Danish trial | 6% | 0.08% |
| Austria | Alfalfa | Danish trial | 12% | 0.07% |
| Belgium | Alfalfa | Danish trial | 12% | 0.09% |
| Denmark | Alfalfa | Danish trial | 12% | 0.09% |
| EU | Alfalfa | Danish trial | 12% | 0.10% |
| Finland | Alfalfa | Danish trial | 12% | 0.08% |
| France | Alfalfa | Danish trial | 12% | 0.08% |
| Germany | Alfalfa | Danish trial | 12% | 0.08% |
| Hungary | Alfalfa | Danish trial | 12% | 0.08% |
| Ireland | Alfalfa | Danish trial | 12% | 0.08% |
| Italy | Alfalfa | Danish trial | 12% | 0.05% |
| Netherlands | Alfalfa | Danish trial | 12% | 0.08% |
| Spain | Alfalfa | Danish trial | 12% | 0.09% |
| Sweden | Alfalfa | Danish trial | 12% | 0.08% |
| Austria | Alfalfa | Danish trial | 24% | 0.08% |
| Belgium | Alfalfa | Danish trial | 24% | 0.10% |
| Denmark | Alfalfa | Danish trial | 24% | 0.11% |
| EU | Alfalfa | Danish trial | 24% | 0.12% |
| Finland | Alfalfa | Danish trial | 24% | 0.09% |
| France | Alfalfa | Danish trial | 24% | 0.10% |
| Germany | Alfalfa | Danish trial | 24% | 0.09% |
| Hungary | Alfalfa | Danish trial | 24% | 0.09% |
| Ireland | Alfalfa | Danish trial | 24% | 0.09% |
| Italy | Alfalfa | Danish trial | 24% | 0.05% |
| Netherlands | Alfalfa | Danish trial | 24% | 0.10% |
| Spain | Alfalfa | Danish trial | 24% | 0.10% |
| Sweden | Alfalfa | Danish trial | 24% | 0.10% |
| Austria | "secure" feed | French dataset | unadjusted | 0.09% |
| Belgium | "secure" feed | French dataset | unadjusted | 0.12% |
| Denmark | "secure" feed | French dataset | unadjusted | 0.12% |
| EU | "secure" feed | French dataset | unadjusted | 0.13% |
| Finland | "secure" feed | French dataset | unadjusted | 0.11% |
| France | "secure" feed | French dataset | unadjusted | 0.11% |
| Germany | "secure" feed | French dataset | unadjusted | 0.10% |
| Hungary | "secure" feed | French dataset | unadjusted | 0.10% |
| Ireland | "secure" feed | French dataset | unadjusted | 0.10% |
| Italy | "secure" feed | French dataset | unadjusted | 0.06% |
| Netherlands | "secure" feed | French dataset | unadjusted | 0.11% |
| Spain | "secure" feed | French dataset | unadjusted | 0.12% |
| Sweden | "secure" feed | French dataset | unadjusted | 0.11% |
| Austria | "secure" feed | French dataset | 6% | 0.10% |
| Belgium | "secure" feed | French dataset | 6% | 0.12% |
| Denmark | "secure" feed | French dataset | 6% | 0.13% |
| EU | "secure" feed | French dataset | 6% | 0.14% |
| Finland | "secure" feed | French dataset | 6% | 0.11% |
| France | "secure" feed | French dataset | 6% | 0.12% |
| Germany | "secure" feed | French dataset | 6% | 0.11% |
| Hungary | "secure" feed | French dataset | 6% | 0.11% |
| Ireland | "secure" feed | French dataset | 6% | 0.11% |
| Italy | "secure" feed | French dataset | 6% | 0.07% |
| Netherlands | "secure" feed | French dataset | 6% | 0.12% |
| Spain | "secure" feed | French dataset | 6% | 0.13% |
| Sweden | "secure" feed | French dataset | 6% | 0.12% |
| Austria | "secure" feed | French dataset | 12% | 0.10% |
| Belgium | "secure" feed | French dataset | 12% | 0.13% |
| Denmark | "secure" feed | French dataset | 12% | 0.14% |
| EU | "secure" feed | French dataset | 12% | 0.15% |
| Finland | "secure" feed | French dataset | 12% | 0.12% |
| France | "secure" feed | French dataset | 12% | 0.13% |
| Germany | "secure" feed | French dataset | 12% | 0.12% |
| Hungary | "secure" feed | French dataset | 12% | 0.11% |
| Ireland | "secure" feed | French dataset | 12% | 0.12% |
| Italy | "secure" feed | French dataset | 12% | 0.07% |
| Netherlands | "secure" feed | French dataset | 12% | 0.13% |
| Spain | "secure" feed | French dataset | 12% | 0.14% |
| Sweden | "secure" feed | French dataset | 12% | 0.13% |
| Austria | "secure" feed | French dataset | 24% | 0.12% |
| Belgium | "secure" feed | French dataset | 24% | 0.15% |
| Denmark | "secure" feed | French dataset | 24% | 0.16% |
| EU | "secure" feed | French dataset | 24% | 0.18% |
| Finland | "secure" feed | French dataset | 24% | 0.14% |
| France | "secure" feed | French dataset | 24% | 0.15% |
| Germany | "secure" feed | French dataset | 24% | 0.13% |
| Hungary | "secure" feed | French dataset | 24% | 0.13% |
| Ireland | "secure" feed | French dataset | 24% | 0.13% |
| Italy | "secure" feed | French dataset | 24% | 0.08% |
| Netherlands | "secure" feed | French dataset | 24% | 0.15% |
| Spain | "secure" feed | French dataset | 24% | 0.16% |
| Sweden | "secure" feed | French dataset | 24% | 0.15% |

Table S. 3. Relative increase in pig production costs in EU countries and in the EU as a result of adopting immunomodulatory interventions

| country | Intervention | Trial/source | Mortality adjustment | Relative increase in total costs |
| --- | --- | --- | --- | --- |
| Austria | FFT | Dutch trial | unadjusted | 6.45% |
| Belgium | FFT | Dutch trial | unadjusted | 8.07% |
| Denmark | FFT | Dutch trial | unadjusted | 8.30% |
| EU | FFT | Dutch trial | unadjusted | 9.07% |
| Finland | FFT | Dutch trial | unadjusted | 7.43% |
| France | FFT | Dutch trial | unadjusted | 7.67% |
| Germany | FFT | Dutch trial | unadjusted | 7.09% |
| Hungary | FFT | Dutch trial | unadjusted | 7.02% |
| Ireland | FFT | Dutch trial | unadjusted | 7.11% |
| Italy | FFT | Dutch trial | unadjusted | 4.45% |
| Netherlands | FFT | Dutch trial | unadjusted | 7.67% |
| Spain | FFT | Dutch trial | unadjusted | 8.17% |
| Sweden | FFT | Dutch trial | unadjusted | 7.65% |
| Austria | FFT | Dutch trial | 6% | 6.84% |
| Belgium | FFT | Dutch trial | 6% | 8.54% |
| Denmark | FFT | Dutch trial | 6% | 8.78% |
| EU | FFT | Dutch trial | 6% | 9.59% |
| Finland | FFT | Dutch trial | 6% | 7.86% |
| France | FFT | Dutch trial | 6% | 8.12% |
| Germany | FFT | Dutch trial | 6% | 7.51% |
| Hungary | FFT | Dutch trial | 6% | 7.43% |
| Ireland | FFT | Dutch trial | 6% | 7.52% |
| Italy | FFT | Dutch trial | 6% | 4.72% |
| Netherlands | FFT | Dutch trial | 6% | 8.12% |
| Spain | FFT | Dutch trial | 6% | 8.64% |
| Sweden | FFT | Dutch trial | 6% | 8.10% |
| Austria | FFT | Dutch trial | 12% | 7.27% |
| Belgium | FFT | Dutch trial | 12% | 9.07% |
| Denmark | FFT | Dutch trial | 12% | 9.32% |
| EU | FFT | Dutch trial | 12% | 10.18% |
| Finland | FFT | Dutch trial | 12% | 8.35% |
| France | FFT | Dutch trial | 12% | 8.63% |
| Germany | FFT | Dutch trial | 12% | 7.98% |
| Hungary | FFT | Dutch trial | 12% | 7.90% |
| Ireland | FFT | Dutch trial | 12% | 8.00% |
| Italy | FFT | Dutch trial | 12% | 5.02% |
| Netherlands | FFT | Dutch trial | 12% | 8.62% |
| Spain | FFT | Dutch trial | 12% | 9.18% |
| Sweden | FFT | Dutch trial | 12% | 8.61% |
| Austria | FFT | Dutch trial | 24% | 8.32% |
| Belgium | FFT | Dutch trial | 24% | 10.36% |
| Denmark | FFT | Dutch trial | 24% | 10.64% |
| EU | FFT | Dutch trial | 24% | 11.60% |
| Finland | FFT | Dutch trial | 24% | 9.55% |
| France | FFT | Dutch trial | 24% | 9.86% |
| Germany | FFT | Dutch trial | 24% | 9.12% |
| Hungary | FFT | Dutch trial | 24% | 9.03% |
| Ireland | FFT | Dutch trial | 24% | 9.14% |
| Italy | FFT | Dutch trial | 24% | 5.77% |
| Netherlands | FFT | Dutch trial | 24% | 9.85% |
| Spain | FFT | Dutch trial | 24% | 10.48% |
| Sweden | FFT | Dutch trial | 24% | 9.83% |
| Austria | E.coli vaccine | French dataset analysis | unadjusted | 0.50% |
| Belgium | E.coli vaccine | French dataset analysis | unadjusted | 0.64% |
| Denmark | E.coli vaccine | French dataset analysis | unadjusted | 0.66% |
| EU | E.coli vaccine | French dataset analysis | unadjusted | 0.73% |
| Finland | E.coli vaccine | French dataset analysis | unadjusted | 0.58% |
| France | E.coli vaccine | French dataset analysis | unadjusted | 0.61% |
| Germany | E.coli vaccine | French dataset analysis | unadjusted | 0.56% |
| Hungary | E.coli vaccine | French dataset analysis | unadjusted | 0.55% |
| Ireland | E.coli vaccine | French dataset analysis | unadjusted | 0.56% |
| Italy | E.coli vaccine | French dataset analysis | unadjusted | 0.34% |
| Netherlands | E.coli vaccine | French dataset analysis | unadjusted | 0.60% |
| Spain | E.coli vaccine | French dataset analysis | unadjusted | 0.65% |
| Sweden | E.coli vaccine | French dataset analysis | unadjusted | 0.60% |
| Austria | E.coli vaccine | French dataset analysis | 6% | 0.53% |
| Belgium | E.coli vaccine | French dataset analysis | 6% | 0.68% |
| Denmark | E.coli vaccine | French dataset analysis | 6% | 0.70% |
| EU | E.coli vaccine | French dataset analysis | 6% | 0.77% |
| Finland | E.coli vaccine | French dataset analysis | 6% | 0.62% |
| France | E.coli vaccine | French dataset analysis | 6% | 0.64% |
| Germany | E.coli vaccine | French dataset analysis | 6% | 0.59% |
| Hungary | E.coli vaccine | French dataset analysis | 6% | 0.58% |
| Ireland | E.coli vaccine | French dataset analysis | 6% | 0.59% |
| Italy | E.coli vaccine | French dataset analysis | 6% | 0.36% |
| Netherlands | E.coli vaccine | French dataset analysis | 6% | 0.64% |
| Spain | E.coli vaccine | French dataset analysis | 6% | 0.69% |
| Sweden | E.coli vaccine | French dataset analysis | 6% | 0.64% |
| Austria | E.coli vaccine | French dataset analysis | 12% | 0.57% |
| Belgium | E.coli vaccine | French dataset analysis | 12% | 0.73% |
| Denmark | E.coli vaccine | French dataset analysis | 12% | 0.75% |
| EU | E.coli vaccine | French dataset analysis | 12% | 0.82% |
| Finland | E.coli vaccine | French dataset analysis | 12% | 0.66% |
| France | E.coli vaccine | French dataset analysis | 12% | 0.69% |
| Germany | E.coli vaccine | French dataset analysis | 12% | 0.63% |
| Hungary | E.coli vaccine | French dataset analysis | 12% | 0.62% |
| Ireland | E.coli vaccine | French dataset analysis | 12% | 0.63% |
| Italy | E.coli vaccine | French dataset analysis | 12% | 0.39% |
| Netherlands | E.coli vaccine | French dataset analysis | 12% | 0.69% |
| Spain | E.coli vaccine | French dataset analysis | 12% | 0.73% |
| Sweden | E.coli vaccine | French dataset analysis | 12% | 0.68% |
| Austria | E.coli vaccine | French dataset analysis | 24% | 0.66% |
| Belgium | E.coli vaccine | French dataset analysis | 24% | 0.84% |
| Denmark | E.coli vaccine | French dataset analysis | 24% | 0.86% |
| EU | E.coli vaccine | French dataset analysis | 24% | 0.95% |
| Finland | E.coli vaccine | French dataset analysis | 24% | 0.77% |
| France | E.coli vaccine | French dataset analysis | 24% | 0.79% |
| Germany | E.coli vaccine | French dataset analysis | 24% | 0.73% |
| Hungary | E.coli vaccine | French dataset analysis | 24% | 0.72% |
| Ireland | E.coli vaccine | French dataset analysis | 24% | 0.73% |
| Italy | E.coli vaccine | French dataset analysis | 24% | 0.45% |
| Netherlands | E.coli vaccine | French dataset analysis | 24% | 0.79% |
| Spain | E.coli vaccine | French dataset analysis | 24% | 0.85% |
| Sweden | E.coli vaccine | French dataset analysis | 24% | 0.79% |
